# Effects of soluble guanylyl cyclase stimulation on muscle oxygenation and exercise capacity in heart failure with mildly reduced ejection fraction

**DOI:** 10.1113/EP092756

**Published:** 2025-06-19

**Authors:** Ramona E. Weber, Kiana M. Schulze, Andrew G. Horn, Tyler E. McCoach, Zachary J. White, K. Sue Hageman, Stephanie E. Hall, Peter Sandner, Brad J. Behnke, Timothy I. Musch, David C. Poole

**Affiliations:** ^1^ Department of Kinesiology Kansas State University Manhattan Kansas USA; ^2^ University of Kansas School of Medicine Kansas City Kansas USA; ^3^ Department of Anatomy & Physiology Kansas State University Manhattan Kansas USA; ^4^ Bayer HealthCare AG, Drug Discovery Wuppertal Germany; ^5^ Institute of Pharmacology, Hannover Medical School Hannover Germany

**Keywords:** cardiovascular disease, cyclic guanosine monophosphate, exercise intolerance, moderate heart failure, nitric oxide, oxygen uptake kinetics

## Abstract

Heart failure (HF) with a mildly reduced ejection fraction (HFmrEF; 40%–49%) is present in ≤25% of HF patients. Therapeutic treatment options for HFmrEF‐associated exercise intolerance are limited. Nitric oxide (NO)‐independent stimulation of soluble guanylyl cyclase to improve peripheral vasodilatation offers a novel approach to enhance skeletal muscle oxygenation (interstitial pressure of O_2_) and exercise capacity. We tested the hypotheses that the soluble guanylyl cyclase stimulator BAY 41‐2272 (BAY41) would increase exercise tolerance, skeletal muscle interstitial pressure of O_2_ during contractions and NO sensitivity in rats with HFmrEF. Healthy Sprague–Dawley rats (*n *= 20) and rats with experimentally induced HFmrEF (*n *= 25) (3–4 months old) were used for this investigation. HFmrEF was confirmed via echocardiography, and rats were randomized and treated with 1.0 mg/kg of BAY41 or vehicle for 2 weeks, followed by exercise testing and spinotrapezius muscle phosphorescence quenching measurements during 1 Hz contractions. The HFmrEF rats had a lower exercise capacity than healthy rats (834 ± 169 vs. 1138 ± 214 s; *p *< 0.001). BAY41 increased exercise capacity (1158 ± 223 s; *p* = 0.006) and muscle oxygenation index (1461 ± 427 vs. 1108 ± 239 mmHg·s; *p* = 0.047) during 1 Hz contractions versus HFmrEF vehicle control rats. Sodium nitroprusside superfusion elicited a faster response time in BAY41‐treated HFmrEF rats versus vehicle‐treated control animals (150 ± 58 vs. 220 ± 50 s; *p* = 0.022). After sodium nitroprusside superfusion, BAY41‐treated HFmrEF rats had a significantly elevated muscle oxygenation index during steady‐state contractions versus vehicle‐treated control animals (3104 ± 703 vs. 2365 ± 682 mmHg·s; *p* = 0.027). These data suggest that stimulation with soluble guanylyl cyclase can improve skeletal muscle oxygenation and increase the exercise capacity in HFmrEF rats, potentially via enhanced vascular smooth muscle NO sensitivity.

## Abstract (highlights)

- **What is the central question of this study?**
  Soluble guanylyl cyclase stimulators are currently used to treat worsening heart failure but might be more effective at increasing exercise capacity in moderate disease states, specifically heart failure with mildly reduced ejection fraction (HFmrEF).
- **What is the main finding and its importance?**
  We measured exercise tolerance during treadmill running and skeletal muscle oxygen delivery‐to‐utilization matching in rats with HFmrEF (ejection fraction 40%–49%) and found that administration of a soluble guanylyl cyclase stimulator improved exercise tolerance and skeletal muscle oxygenation during contractions owing, in part, to increased sensitivity to nitric oxide donation.

## 1 INTRODUCTION

Heart failure (HF) imposes an extensive healthcare burden afflicting ∼64 million people worldwide and is classified into three subtypes based on left ventricular (LV) ejection fraction (EF): HF with preserved EF (HFpEF), HF with reduced EF (HFrEF) and HF with mildly reduced ejection fraction EF (HFmrEF) (Bozkurt et al., 2021; Savarese et al., 2023). It is estimated that HFmrEF makes up to ∼25% of the clinical HF population (Hsu et al., 2017). HFmrEF patients share several characteristics with the HFrEF phenotype, including male sex, younger age and ischaemic aetiology, but are less symptomatic [lower New York Heart Association (NYHA) class] and have fewer comorbidities than either HFrEF or HFpEF patients (Chioncel et al., 2017). Despite this, these individuals have a significantly reduced peak oxygen uptake (${\dot{V}}_{O_{2}\mathrm{peak}}$) during exercise of ∼17 mL/kg/min (range: ∼12–21 mL/kg/min) (Pugliese et al., 2019). The ${\dot{V}}_{O_{2}\mathrm{peak}}$ stratifies risk for cardiac transplant, clinical trial inclusion criteria and innovative pharmacotherapy for all degrees of HF (Parikh et al., 2009); however, HFmrEF (EF of 40%–49%) is frequently excluded from clinical trials that instead focus on severe HF (EF of <40%; NYHA Class III–IV) (Savarese et al., 2022). This poses a significant challenge for individuals with HFmrEF, because timely initiation of guideline‐directed medical therapy is crucial. One promising target is the nitric oxide (NO)‐soluble guanylyl cyclase (sGC)–cyclic guanosine monophosphate (cGMP) signalling pathway, which could potentially be leveraged to improve skeletal muscle O_2_ transport and impede disease progression in HFmrEF.

In HF, reduced NO bioavailability and impaired NO‐mediated vasodilatation (Katz et al., 1996; Kiowski et al., 1998; Kubo et al., 1991) underlie, in part, the microvascular O_2_ supply (${\dot{Q}}_{O_{2}}$) response, which is inadequate to match metabolic demand (${\dot{V}}_{O_{2}}$) in skeletal muscle at the onset of exercise (Poole et al., 2012). Consequently, the partial pressure of O_2_ within the interstitial space ($P_{O_{2}\mathrm{is}}$), which is directly reflective of ${\dot{Q}}_{O_{2}}$‐to‐${\dot{V}}_{O_{2}}$ matching, decreases with worsening HF (slower ${\dot{V}}_{O_{2}}$ kinetics, increased O_2_ delivery dependence) (Craig et al., 2019). Targeting the downstream NO receptor, sGC, has been shown to increase exercise tolerance in humans and in animal models of cardiovascular disease (Ghofrani et al., 2013; Hu et al., 2023); however, the effects on peripheral O_2_ transport are not known.

The NO–sGC system plays an important role in the regulation of vascular tone by increasing cGMP to promote smooth muscle relaxation (Derbyshire & Marletta, 2012). The spectrum of oxidative stress across HF severity reduces sGC sensitivity to upstream NO (Derbyshire & Marletta, 2012) and/or renders sGC inactive via oxidation (Stasch & Hobbs, 2009), thereby disrupting NO–cGMP signalling. Therefore, the discovery of NO‐independent sGC stimulators was a landmark in targeting and enhancing cGMP signalling (Sandner et al., 2021). These compounds, such as Vericiguat, stimulate sGC in the absence of NO, increase sGC sensitivity and have a synergistic effect with endogenous NO (Breitenstein et al., 2017; Sandner et al., 2021). Unfortunately, sGC stimulation in patients with worsening HFrEF did not improve exercise capacity (Armstrong et al., 2020). A secondary analysis revealed that sGC stimulators were more effective at reducing all‐cause mortality in stable HF, which suggests that targeting sGC in HFmrEF might be efficacious (Butler et al., 2022). Therefore, the aim of the present study was to investigate the effects sGC stimulation on skeletal muscle oxygenation ($P_{O_{2}\mathrm{is}}$) and exercise tolerance in rats with HFmrEF. We tested the hypotheses that, compared with vehicle control rats, HFmrEF rats treated with the sGC stimulator BAY 41‐2272 (BAY41) would have: (1) enhanced $P_{O_{2}\mathrm{is}}$ kinetics during contractions (i.e., slower $P_{O_{2}\mathrm{is}}$ fall from rest to contractions); (2) increased sensitivity to exogenous NO (i.e., accelerated rise in $P_{O_{2}\mathrm{is}}$); and (3) an improved exercise capacity.

## 2 MATERIALS AND METHODS

### 2.1 Ethical approval

**FIGURE 1:**
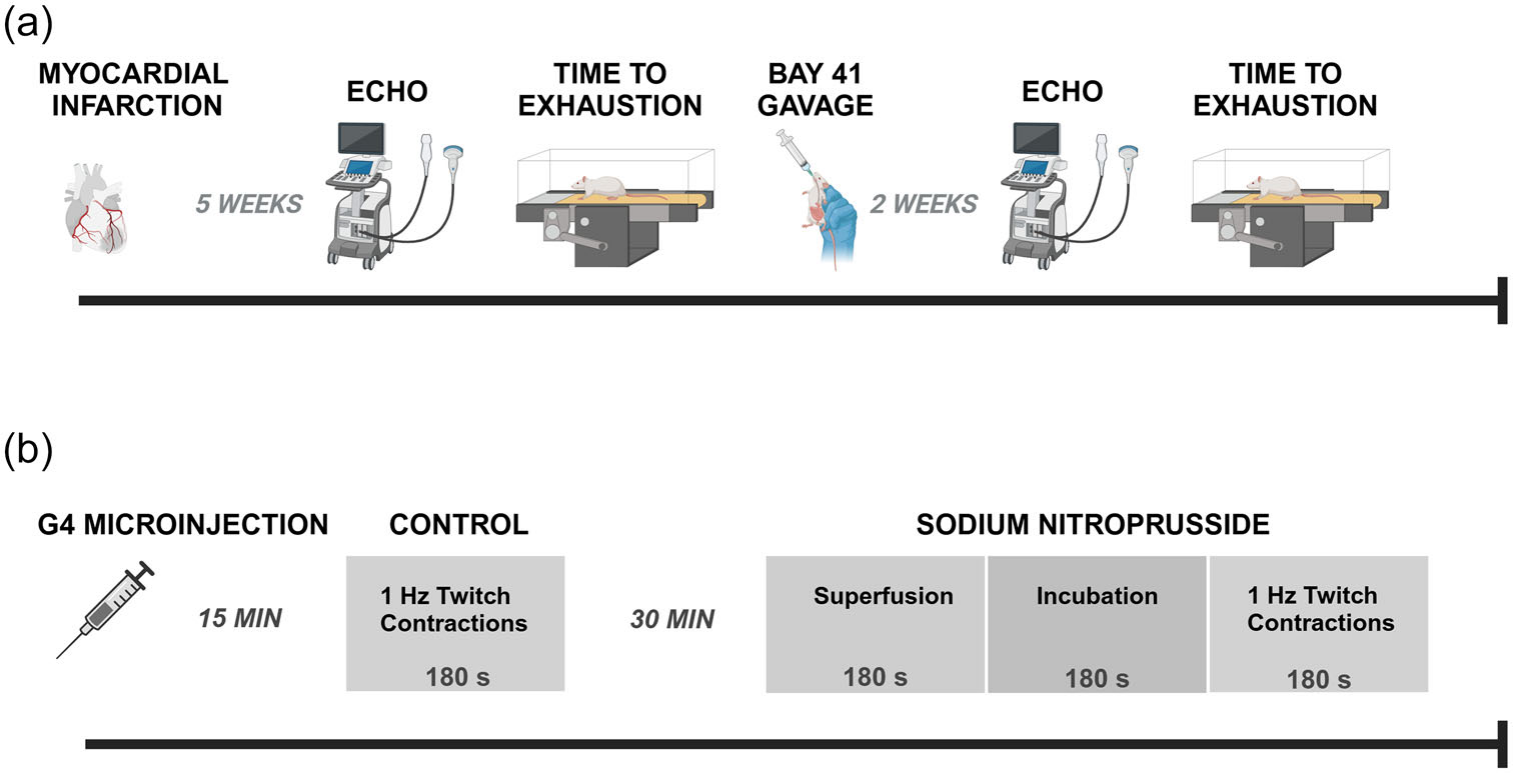
Experimental protocol for exercise measurements, dosing regimen and echocardiography (a) and phosphorescence quenching measurements (b). Created in BioRender. https://BioRender.com/vynpu76. Abbreviations: echo, echocardiogram; BAY 41, BAY 41‐2272.

All procedures were approved by the Institutional Animal Care and Use Committee of Kansas State University (protocol #4719), conformed to the *Guide for the Care and Use of Laboratory Animals* published by the US National Institutes of Health (NIH publication no. 85‐23, revised 1985) and were conducted according to the National Research Council Guide for the Care and Use of Laboratory Animals. Experiments were performed on male Sprague–Dawley rats (*n *= 45; 3–4 months old; Charles River Laboratories, Wilmington, MA, USA). Upon arrival, animals were maintained in accredited (Association for the Assessment and Accreditation of Laboratory and Animal Care, 00667; Office of Laboratory Animal Welfare, A3609‐01) animal facilities under a 12 h–12 h light–dark cycle with food and water provided ad libitum. The experimental design for both the non‐invasive and invasive measurements is presented in Figure 1.

### 2.2 Myocardial infarction procedure

Rats were randomized to either Healthy (*n *= 20) or HFmrEF (*n *= 25) groups. HFmrEF was induced via surgical ligation of the left main coronary artery [i.e., myocardial infarction (MI)]. Immediately prior to surgery, HFmrEF rats were administered amiodarone (110 mg/kg i.p.; Fresenius Kabi, Lake Zurich, IL, USA) to reduce the development of ventricular arrhythmias during the procedure. Rats were anaesthetized with a 2.5% isoflurane–O_2_ (Butler Health Supply, Dublin, OH, USA) mixture and intubated for mechanical ventilation with a rodent respirator (model 680, Harvard Apparatus, Holliston, MA, USA) for the duration of the surgical procedure. The heart was then exposed via a thoracotomy at the fifth intercostal space, and the left main coronary artery was ligated with 6–0 silk suture ∼1–2 mm distal to the edge of the left atrium. The incision was closed, and rats were administered ampicillin (50 mg/kg i.m.; Fresenius Kabi, Lake Zurich, IL, USA) to reduce susceptibility to infection and analgesic agents [bupivacaine (1.5 mg/kg s.c.; Hospira, Lake Forest, IL, USA) and buprenorphine (0.01–0.05 mg/kg i.m.; Par Pharmaceutical, Chestnut Ridge, NY, USA)]. Rats were monitored for ∼4 h post‐surgery for arrhythmia and signs of stress. An intensive 14 day post‐operative plan ensured daily assessment (for reduced appetite, weight loss or changes in gait/posture). Our laboratory has demonstrated ∼70% success with this model, where some animals display preserved EF (0% MI size, no remodelling) or experience arrhythmias following surgery that exacerbate myocardial remodelling and severely reduce EF (EF < 30%).

### 2.3 Echocardiography

Five weeks after the MI procedure, LV function was assessed via transthoracic echocardiography (see Figure 1a) using a commercially available system (Logiq S8; GE Health Care, Milwaukee, WI, USA) with an 18 MHz linear transducer (L8‐18i) as previously described (Craig et al., 2019). Rats were initially anaesthetized with a 5% isoflurane–O_2_ mixture (and maintained on <2.5% isoflurane–O_2_ (Butler Health Supply, Doblin, OH, USA) while positioned on a heating pad to keep core temperature at ∼37°C (measured via rectal thermometer). Standard two‐dimensional and M‐mode images from the mid‐papillary level were obtained with frame rates of >50 frames/s. Ventricular dimensions and wall thicknesses were obtained from M‐mode measurements over four consecutive cardiac cycles. Measurements presented are an average of the four cardiac cycles. LV internal dimensions (ID, in centimetres) and posterior wall (PW, in centimetres) thickness were measured at end systole (LVIDs, in centimetres; PWs, in centimetres) and end diastole (LVIDd, in centimetres; PWd, in centimetres). Fractional shortening (FS, as a percentage) was calculated from the measurements of LV chamber diameters: FS = [(LVIDd − LVIDs)/LVIDd] × 100. LV end‐systolic (LVESV, in millilitres) and end‐diastolic (LVEDV, in millilitres) volumes were calculated using the Teicholz formula: LV volume = (7.0/2.4 + LV dimension) × LV dimension^3^. Stroke volume was calculated as follows: SV = LVEDV − LVESV. Ejection fraction (EF, as a percentage) was calculated as follows: EF = [(LVEDV − LVESV)/LVEDV]  × 100.

### 2.4 Treadmill acclimation and time to exhaustion

After the initial echocardiogram, all healthy (*n *= 20) or HFmrEF (*n *= 25) rats were familiarized with running on a motor‐driven treadmill for 5 min/day for 5 days at 25 m/min up a 5% gradient. After treadmill familiarization, rats performed a time‐to‐exhaustion test to assess exercise capacity (Copp et al., 2009; Poole et al., 2020). All exercise tests were performed between 08.00 h and 16.00 h to diminish diurnal variation in the results (Clark & Conlee, 1979). Throughout the exercise protocols, the room temperature was maintained at ∼21°C–22°C. The time‐to‐exhaustion protocol consisted of a progressive exercise test, in which rats began at a speed of 25 m/min up a 5% gradient for 15 min. Thereafter, the speed increased by 5 m/min every 15 min until the rat was unable or unwilling to maintain pace with the treadmill belt despite encouragement by manual bursts of high‐pressure air aimed at the hindlimbs. Fatigue was preceded by a lowering of the hindquarters and a raised snout, which resulted in a significantly altered gait. Time from the start to the cessation of exercise was measured and recorded to the nearest second. Exhaustion was confirmed by placing the rat in a supine position and observing absence or a delay of the righting reflex (Poole et al., 2020). This protocol typically induces exhaustion within 1500 s, concomitant with running constituting severe intensity exercise (i.e., above critical speed) (Copp et al., 2009, 2010). Rats were weighed upon completion of the test, and the results reflect the end‐exercise weight.

### 2.5 Drug dosing

After initial time‐to‐exhaustion protocols, Healthy and HFmrEF rats were assigned randomly to sGC stimulator (Healthy + BAY41, *n *= 10; HFmrEF + BAY41, *n *= 15) or vehicle control (Healthy, *n *= 10; HFmrEF, *n *= 10) treatment groups. Eight HFmrEF rats (HFmrEF + BAY41, *n *= 5; HFmrEF, *n *= 3) were excluded owing to EF outside the 40%–50% range or no evidence of MI (0% infarct size), resulting in a final sample size of *n *= 7 for HFmrEF vehicle controls and *n *= 10 for HFmrEF + BAY41. The sGC stimulator BAY 41‐2272 {BAY41; 5‐cyclopropyl‐2‐[1‐(2‐fluorobenzyl)‐1*H*‐pyrazolo[3,4‐b]pyridin‐3‐yl]‐pyrimidin‐4‐ylamine; Bayer Pharmaceuticals, Wuppertal, Germany} was weighed and mixed with a vehicle that consisted of 10% Transcutol (diethylene glycol monoethyl ether; Sigma–Aldrich, St. Louis, MO, USA), 20% Cremophor EL (Sigma–Aldrich, St. Louis, MO, USA) and 70% water to obtain a dose of 1.0 mg/kg BAY41 in 1 mL of vehicle. This dose accounts for the ∼6‐ to 7‐fold greater resting metabolic rate in rats compared with humans (Poole et al., 2020). BAY41 or vehicle was administered via oral gavage, detailed previously (Weber et al., 2022), twice daily for 14 days. The day after the final dose, we performed the same protocol detailed above (see Sections 2.3 and 2.4).

### 2.6 Surgical preparations and phosphorescence quenching

Twenty‐four hours after the final time‐to‐exhaustion measurements, rats were anaesthetized with a 5% isoflurane–O_2_ mixture and maintained with 2.5% isoflurane–O_2_ while core temperature was kept at ∼37°C (measured via rectal thermometer). The left carotid artery was isolated, cannulated and a 2 Fr catheter‐tip pressure transducer (Millar Instruments, Houston, TX, USA) was advanced into the LV to measure LV end‐diastolic pressure (LVEDP) and changes in LV pressure over time (LV d*P*/d*t*). After the Millar measurements, the transducer was removed and the carotid artery was cannulated with PE‐10 connected to PE‐50 (Intra‐Medic polyethylene tubing; Clay Adams Brand, Benton, Dickson and Company, Sparks, MD, USA) to measure mean arterial pressure and heart rate (Digi‐Med BPA; model 400). A second catheter was placed in the caudal artery for administration of pentobarbitone sodium anaesthesia (50 mg/mL) and arterial blood sampling. Rats were then transitioned to pentobarbitone sodium anaesthesia (20 mg/kg body weight i.a.) while the concentration of isoflurane was decreased and subsequently discontinued. The depth of anaesthesia was monitored regularly via toe pinch and palpebral reflex, with pentobarbitone anaesthesia supplemented (3.5–7.0 mg/kg) as necessary during experimentation.

To exteriorize the spinotrapezius muscle, the overlying skin and fascia were carefully removed from the mid‐dorsal–caudal region, and the spinotrapezius muscle was exposed. Importantly, this exteriorization does not damage the vascular supply or neural innervation to the muscle (Bailey et al., 2000; Gray, 1973; Kindig et al., 1999). This muscle was chosen for its similar fibre‐type composition and oxidative capacity to the untrained human quadriceps muscle (Delp & Duan, 1996). Silver wire electrodes were sutured (6–0 silk) to the rostral (cathode) and caudal (anode) regions of the muscle to induce twitch contractions. The muscle was continuously superfused with Krebs–Henseleit bicarbonate‐buffered solution (mM: 4.7 KCl, 2.0 CaCl_2_, 2.4 MgSO_4_, 131 NaCl and 22 NaHCO_3_; pH = 7.40; equilibrated with 5% CO_2_–95% N_2_ at 38°C), and the adjacent tissue was covered with Saran wrap (S.C. Johnson & Son, Racine, WI, USA) to minimize dehydration.

Figure 1b describes the experimental protocol herein. All the following measurements were completed in a dark room to minimize interference with the phosphorescence signal. Oxyphor G4 [Pd‐meso‐tetra‐(3,5‐dicarboxyphenyl)‐tetrabenzo‐porphyrin], a phosphorescent probe that permits the dynamic visualization of tissue $P_{O_{2}}$ (Esipova et al., 2011), was injected locally into the muscle (three to four injections, 10 µL in total at 10 µM) using a 29‐gauge needle. Care was taken to avoid damaging any visible vasculature. Following G4 injections, the spinotrapezius muscle was covered with Saran wrap to minimize dehydration during a 15 min period that allows for diffusion of Oxyphor G4 throughout the muscle and into the interstitial space. A bifurcated light guide was positioned ∼3–4 mm above the surface of the exposed muscle in a field absent of large vessels.

Thereafter, a frequency‐domain phosphorimeter (PMOD 500; Oxygen Enterprises, Philadelphia, PA, USA) was used as previously described (Craig et al., 2018) to determine resting and contracting $P_{O_{2}\mathrm{is}}$ during two separate conditions: control (CON) and sodium nitroprusside superfusion (SNP, 300 µM). Twitch contractions were evoked electrically (1 Hz, 6 V, 2 ms pulse duration) with a Grass S88 Stimulator (Quincy, MA, USA) for 180 s, and $P_{O_{2}\mathrm{is}}$ was measured and recorded at 2 s intervals throughout the duration of the protocol. This stimulation protocol elicits a ∼4‐ to 5‐fold increase in ${\dot{Q}}_{O_{2}}$ and a ∼7‐fold increase in ${\dot{V}}_{O_{2}}$ that is consistent with moderate‐intensity exercise (Behnke et al., 2001; Hirai et al., 2013). Immediately following CON $P_{O_{2}\mathrm{is}}$ measurements, 0.3 mL of blood was sampled from the caudal artery catheter for the analysis of arterial blood gases. After 30 min of recovery, $P_{O_{2}\mathrm{is}}$ was measured briefly (∼30 s) to verify a return to baseline values. Thereafter, the spinotrapezius was superfused with SNP for 180 s, and $P_{O_{2}\mathrm{is}}$ was recorded continuously. Once a stable $P_{O_{2}\mathrm{is}}$ was confirmed (additional 180 s), the same stimulation protocol as above was repeated in the presence of SNP. Following the last contraction protocol, rats were killed via pentobarbitone sodium (>100 mg/kg) overdose and cardiac excision. The MI size (percentage infarct) was determined by planimetry as previously described (Craig et al., 2019; Pfeffer et al., 1979).

### 2.7 Analysis of interstitial $P_{O_{2}}$ kinetics

The kinetics analysis of the $P_{O_{2}\mathrm{is}}$ response was measured using the Stern–Volmer relationship. Direct measurement of phosphorescence lifetime yielded $P_{O_{2}\mathrm{is}}$ via the following equation:

$$P_{O_{2}}=\left(\frac{\tau^{\circ }}{\tau -1}\right)/\left(k_{Q}\times \tau^{\circ }\right)$$

where *k*_Q_ is the quenching constant and τ and τ° are the phosphorescence lifetimes at the ambient O_2_ concentration and in the absence of O_2_, respectively. In tissues at 32.3°C (muscle surface temperature ∼32°C), the parameters for G4 were as follows: *k*_Q_ of 258 mmHg/s and τ° of 226 µs. Muscle temperature does not change appreciably during the contraction protocol herein; therefore, the phosphorescence lifetime is affected exclusively by the $P_{O_{2}\mathrm{is}}$. The kinetics analyses of the $P_{O_{2}\mathrm{is}}$ response during contractions were modelled with $P_{O_{2}\mathrm{is}}$ measurements collected during 30 s of rest and 180 s of contractions and with either a one‐ or two‐component model:

One component:

$$P_{O_{2}}\left(t\right)=P_{O_{2}\left(\mathrm{BL}\right)}-\Delta_{1}P_{O_{2}}\left[1-e^{-\left(t-TD_{1}/\tau_{1}\right)}\right]$$

Two component:

$$P_{O_{2}}\left(t\right)=P_{O_{2}\left(\mathrm{BL}\right)}-\Delta_{1}P_{O_{2}}\left[1-e^{-\left(t-TD_{1}/\tau_{1}\right)}\right]+\Delta_{2}P_{O_{2}}\left[1-e^{-\left(t-TD_{2}/\tau_{2}\right)}\right]$$

The SNP superfusion response was fitted with $P_{O_{2}\mathrm{is}}$ at rest (30 s) and during 180 s of SNP superfusion:

SNP superfusion:

$$P_{O_{2}}\left(t\right)=P_{O_{2}\left(\mathrm{BL}\right)}+\Delta_{1}P_{O_{2}}\left[1-e^{-\left(t-TD_{1}/\tau_{1}\right)}\right]$$

where $P_{O_{2}}\left(t\right)$ represents the $P_{O_{2}\mathrm{is}}$ at any given timepoint, $P_{O_{2}\left(\mathrm{BL}\right)}$ corresponds to the 30 s of resting $P_{O_{2}\mathrm{is}}$ before contractions, $\Delta_{1}P_{O_{2}}$ and $\Delta_{2}P_{O_{2}}$ are the amplitude for the first and second components, respectively, TD_1_ and TD_2_ are the time delays for the first and second component, respectively, and τ_1_ and τ_2_ are the time constants (i.e., the time required to reach 63% of the amplitude) for each component. Appropriate fits were determined by: (1) the coefficient of determination; (2) the sum of the squared residuals; and (3) visual inspection and analysis of the model fit to the data and residuals. The mean response time (MRT) is the overall kinetics of the primary response during contractions or SNP superfusion (Macdonald et al., 1997):

$$\mathrm{MRT}\;=\;TD_{1}+\tau_{1}$$

where TD_1_ and τ_1_ are defined above. When a secondary component was present, the primary amplitude was constrained not to exceed the nadir value, in order to maximize the accuracy of the primary response kinetics. Given that the second amplitude ($\Delta_{2}P_{O_{2}}$, undershoot of the $P_{O_{2}\mathrm{is}}$) was often mono‐exponential in nature, it was calculated manually by subtracting the difference between the $P_{O_{2}\mathrm{is}}$ at the end of contractions minus the nadir value of $P_{O_{2}\mathrm{is}}$ during contractions. The time taken to reach 63% of the response was determined independently of modelling procedures (*T*_63_). Area under the curve (AUC, in millimetres of mercury times seconds) was integrated by summing each 2 s value across the 180 s of contractions or superfusion to provide an index of overall spinotrapezius muscle interstitial oxygenation.

### 2.8 Western immunoblotting

In a subset of animals (Healthy, *n *= 7; Healthy + BAY41, *n *= 7; HFmrEF, *n *= 7; HFmrEF + BAY41, *n *= 7), 20 mg portions of spinotrapezius muscle samples were homogenized in radio‐immune precipitation assay (RIPA) buffer (Sigma–Aldrich) containing a protease and phosphatase inhibitor (Pierce Protease and Phosphatase Inhibitor Mini Tablet; ThermoFisher Scientific) prepared according to the manufacturer's guidelines. Lysate protein concentrations were determined using the Qubit 4 Fluorometer with Protein Broad Range Assay kit (ThermoFisher Scientific). Protein quantification in medial costal diaphragm lysates was completed with Jess Simple Western (ProteinSimple) automated western blot analysis with primary antibodies for soluble guanylyl cyclase subunit beta 1 (sGCβ1; NBP1‐89784, Novus Biologicals, 1:10 dilution) and superoxide dismutase 1 (SOD1; NBP1‐90186, Novus Biologicals, 1:100 dilution). Lysate protein concentrations were equalized (1 mg/mL) before protein quantification, and each target protein was assessed in a single trial to ensure equivalent primary antibody exposure across spinotrapezius lysates, thus removing the need for a housekeeping protein. Protein expression was quantified with Compass for Simple Western software.

### 2.9 Statistical analysis

Statistical analyses and curve fitting were performed using a commercially available software package (SigmaPlot v.12.5; Systat Software, San Jose, CA, USA). Two‐way ANOVA tests were performed to determine differences in time to exhaustion, morphological characteristics, LV echocardiography, Millar LV pressures and protein expression, and to detect differences present in $P_{O_{2}\mathrm{is}}$ kinetics parameters. A two‐way repeated‐measures ANOVA evaluated temporal interactions (group × time) during $P_{O_{2}\mathrm{is}}$ measurements. Normality was assessed using the Shapiro–Wilk test. If either the normality or equal variance assumptions were violated, a Mann–Whitney rank‐sum test was used to compare the above variables. Tukey's *post hoc* tests were used for multiple comparisons when significant differences were detected. If data sets were missing values, statistical analysis was done using a two‐way mixed ANOVA test, followed by *post hoc* pairwise comparisons using Fisher's least significant difference test without correction for multiple comparisons to preserve statistical power. Data are presented as the group means ± SD. Significance was accepted at *p *< 0.05.

## 3 RESULTS

### 3.1 Echocardiographic and morphometric data

At 5 weeks, all Healthy rats (*n *= 20) displayed normal LV function (LVEF ∼80%), whereas 17 of 25 MI rats displayed HFmrEF (LVEF between 40% and 49%). Eight HFmrEF rats (HFmrEF + BAY, *n *= 5; HFmrEF, *n *= 3) were excluded owing to EF outside the 40%–50% range or no evidence of MI (0% infarct size). No differences in LV function were present within groups prior to randomization into vehicle control or BAY41 groups (5 weeks). At 7 weeks post‐MI, 37 rats (Healthy, *n *= 10; Healthy + BAY41, *n *= 10; HFmrEF, *n *= 7; HFmrEF + BAY41, *n *= 10) were analysed for haemodynamic and morphometric data. No differences in body weight were present across groups (Healthy, 503 ± 76 g and Healthy + BAY41, 468 ± 83 g, *p* = 0.401; HFmrEF, 513 ± 48 g and HFmrEF + BAY41, 486 ± 30 g, *p* = 0.226), LV mass (Healthy, 1.02 ± 0.07 g and Healthy + BAY41, 1.05 ± 0.07 g, *p* = 0.379; HFmrEF, 1.03 ± 0.07 g and HFmrEF + BAY41, 1.05 ± 0.20 g,  *p* = 0.753) or lung weight (Healthy, 1.61 ± 0.16 g and Healthy + BAY41, 1.75 ± 0.20 g, *p* = 0.131; HFmrEF, 1.74 ± 0.10 and HFmrEF + BAY41, 1.79 ± 0.29 g, *p* = 0.634). LV echocardiographic measurements following the dosing regimen (at 7 weeks post‐MI) are presented in Table 1. The ∼2‐ to 2.5‐fold increase in LVEDV in HFmrEF rats was compensated for by a reduction in FS and EF, such that SV was unchanged in HFmrEF rats. Despite significantly higher LVEDP (16 ± 3 vs. 13 ± 2 mmHg; *p* = 0.005) and lower LV d*P*/d*t* (6300 ± 1142 vs. 7359 ± 1034 mmHg/s; *p* = 0.021) in HFmrEF versus Healthy rats, respectively, no statistical differences in Millar catheter measurements were detected between treated and untreated conditions within HFmrEF (LVEDP, *p* = 0.218; LVd*P*/d*t*, *p* = 0.341) or Healthy (LVEDP, *p* = 0.115; LV d*P*/d*t*, *p* = 0.846) groups. These data and the index of myocardial damage (∼30%) indicate moderate HF within HFmrEF groups.

**TABLE 1:** Left ventricle echocardiography and pressure measurements.

Parameter	Healthy	Healthy + BAY41	*p*‐Value	HFmrEF	HFmrEF + BAY41	*p*‐Value
	(*n *= 10)	(*n *= 10)		(*n *= 7)	(*n *= 10)	
LVIDd, cm	0.66 ± 0.06	0.66 ± 0.12	0.873	0.92 ± 0.15*	0.91 ± 0.12*	0.594
LVIDs, cm	0.31 ± 0.05	0.35 ± 0.11	0.401	0.74 ± 0.12*	0.73 ± 0.09*	0.856
LVPWd, cm	0.25 ± 0.05	0.28 ± 0.11	0.683	0.24 ± 0.06	0.25 ± 0.07	0.765
LVPWs, cm	0.36 ± 0.06	0.39 ± 0.07	0.641	0.31 ± 0.06	0.36 ± 0.10	0.329
FS, %	52 ± 5	48 ± 9	0.513	19 ± 4*	20 ± 5*	0.289
LVEDV, mL	0.67 ± 0.16	0.70 ± 0.37	0.822	1.73 ± 0.69*	1.60 ± 0.41*	0.648
LVESV, mL	0.09 ± 0.04	0.14 ± 0.12	0.557	0.97 ± 0.37*	0.86 ± 0.23*	0.524
SV, mL	0.58 ± 0.13	0.57 ± 0.26	0.606	0.76 ± 0.33	0.74 ± 0.22	0.718
EF, %	87 ± 4	83 ± 9	0.636	43 ± 3*	42 ± 5*	0.646
MI size, %	–	–	–	30 ± 5	30 ± 8	0.904
LVEDP, mmHg	12 ± 2	11 ± 3	0.503	15 ± 2	17 ± 2	0.218
LV d*P*/d*t*, mmHg/s	7250 ± 544	7416 ± 1559	0.846	6581 ± 1234	6018 ± 1044	0.341

*Note*: Data are means ± SD. *n*, number of rats. Abbreviations: BAY41, BAY 41‐2272; EF, ejection fraction; FS, fractional shortening; HFmrEF, heart failure with mildly reduced ejection fraction; LV, left ventricle; LV d*P*/d*t*, LV change in pressure over change in time; LVEDP, LV end‐diastolic pressure; LVEDV, LV end‐diastolic volume; LVESV, LV end‐systolic volume; LVIDd, LV internal diameter in diastole; LVIDs, LV internal diameter in systole; LVPWd, LV posterior wall in diastole; LVPWs, LV posterior wall in systole; MI, myocardial infarction; SV, stroke volume. *p < 0.05 versus Healthy, analysed via two‐way ANOVA with Tukey's *post hoc* analyses.

### 3.2 Time to exhaustion

**FIGURE 2:**
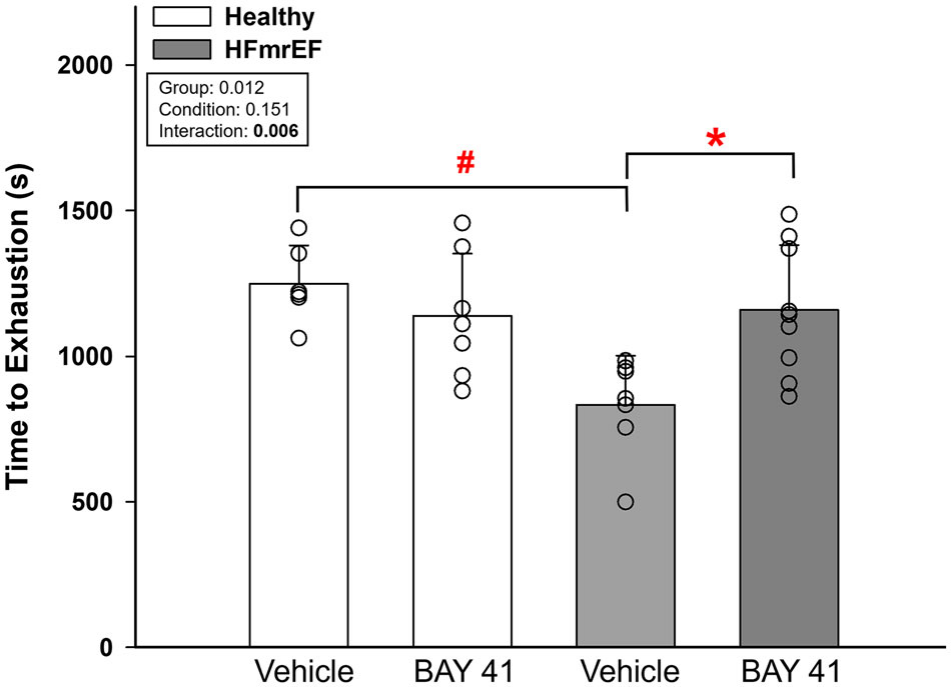
Average and individually plotted time to exhaustion. Data are means ± SD. Healthy rats (*n *= 6), Healthy + BAY41 (*n *= 7), HFmrEF (*n *= 7) and HFmrEF + BAY41 (*n *= 9). Data were analysed via two‐way ANOVA with Tukey's *post hoc* analyses. ^#^ *p *< 0.001 versus Healthy; *p = 0.006 versus HFmrEF Vehicle. Abbreviations: BAY41, BAY 41‐2272; HFmrEF, heart failure with mildly reduced ejection fraction.

Twenty‐nine rats (Healthy, *n *= 6; Healthy + BAY41, *n *= 7; HFmrEF, *n *= 7; HFmrEF + BAY41, *n *= 9) that ran for <1500 s (indicating that the rats reached severe‐intensity exercise) were analysed for time‐to‐exhaustion protocols. There were no differences in time to exhaustion prior to drug/vehicle randomization within Healthy (1349 ± 128 vs. 1395 ± 312 s, *p* = 0.781) or HFmrEF (1016 ± 286 vs. 1073 ± 242 s, *p* = 0.708) groups. Time to exhaustion was greater in HFmrEF + BAY41 rats (1158 ± 223 s) in comparison to HFmrEF rats (834 ± 169 s; *p* = 0.006) such that there were no differences in time to exhaustion between Healthy, Healthy + BAY41 and HFmrEF + BAY41 rats (Healthy vs. Healthy + BAY41, *p* = 0.285; Healthy vs. HFmrEF + BAY41, *p* = 0.397) (Figure 2).

### 3.3 Arterial blood gases and haemodynamics

No differences in heart rate or mean arterial pressure between Healthy and Healthy + BAY41 or HFmrEF and HFmrEF + BAY41 rats were present during CON and SNP protocols (*p *> 0.05). Arterial $P_{O_{2}}$ was reduced in HFmrEF + BAY41 rats compared with HFmrEF rats (86.1 ± 8.2 vs. 95.6 ± 10.9 mmHg, *p* = 0.044), whereas O_2_ saturation was not significantly different (*p* = 0.189). No statistical differences in pH, CO_2_, O_2_ saturation, haematocrit or lactate were present among groups (*p *> 0.05).

### 3.4 Control interstitial $P_{O_{2}}$ kinetics

**FIGURE 3:**
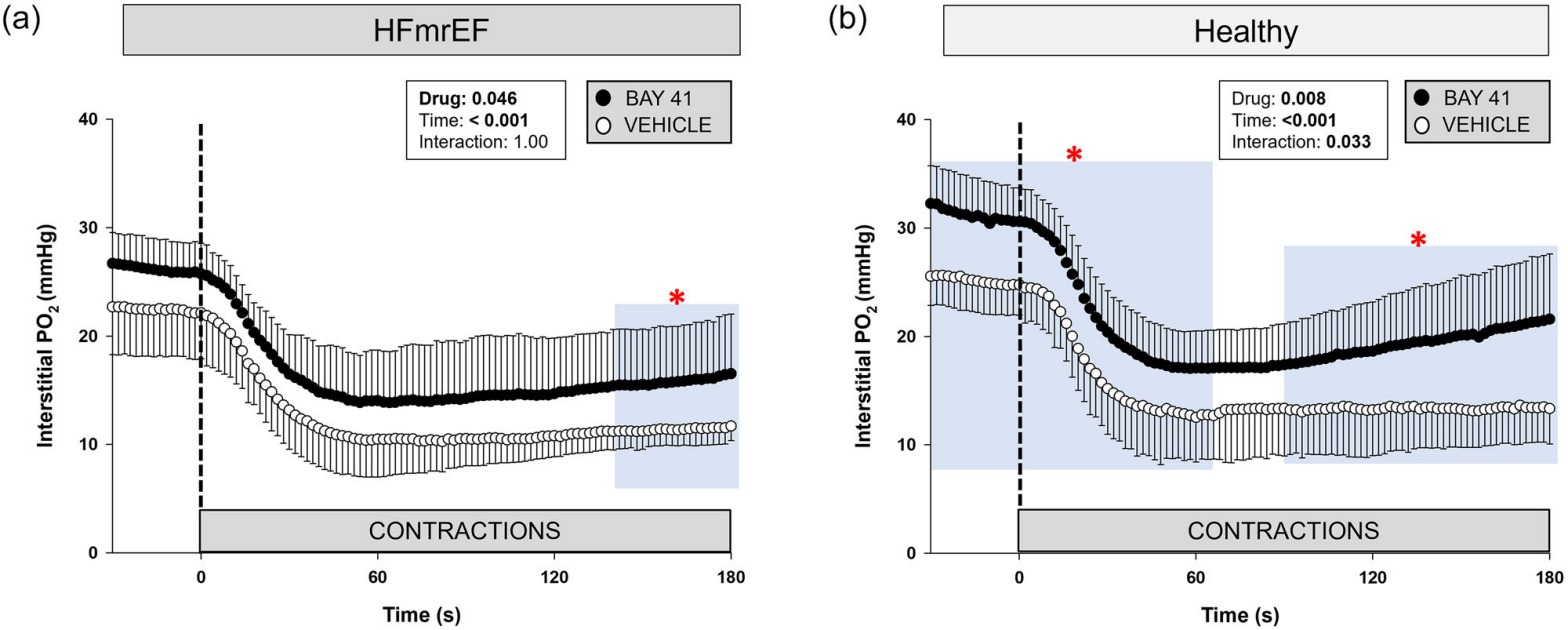
Spinotrapezius muscle interstitial $P_{O_{2}}$ response at rest (from −30 to 0 s) and during twitch contractions (0–180 s) for Healthy rats (*n *= 8), Healthy + BAY41 (*n *= 8), HFmrEF (*n *= 7) and HFmrEF + BAY41 (*n *= 10). Data are means ± SD. The dashed line represents the onset of twitch contractions. (a) HFmrEF + BAY41 rats had a significantly higher $P_{O_{2}\mathrm{is}}$ during steady‐state contractions and from 140 to 180 s (blue area) versus HFmrEF vehicle control rats. (b) Healthy + BAY41 rats had a significantly greater $P_{O_{2}\mathrm{is}}$ at rest (from −30 to 0 s) and during twitch contractions (30–60 and 90–180 s) (blue areas). The $P_{O_{2}\mathrm{is}}$ was compared across time via two‐way repeated‐measures ANOVA with Tukey's *post hoc* analyses. *p < 0.05 for each 2 s increment versus Vehicle within group. Abbreviations: BAY41, BAY 41‐2272; HFmrEF, heart failure with mildly reduced ejection fraction; $P_{O_{2}\mathrm{is}}$, interstitial $P_{O_{2}}$.

Thirty‐three rats (Healthy, *n *= 8; Healthy + BAY41, *n *= 8; HFmrEF, *n *= 7; HFmrEF + BAY41, *n *= 10) were analysed for CON $P_{O_{2}\mathrm{is}}$ kinetics. Four Healthy rats were excluded from the data set owing to haemodynamic complications during/after catheterization. Four Healthy rats, seven Healthy + BAY41, four HFmrEF and nine HFmrEF + BAY41 exhibited profiles that fell transiently below the contracting steady state (i.e., undershoot, $\Delta_{2}P_{O_{2}}$), necessitating a two‐component model fit. The $P_{O_{2}\mathrm{is}}$ kinetics and nadir between HFmrEF and HFmrEF + BAY41 rats were not different (Table 2), but HFmrEF + BAY41 rats had a higher baseline $P_{O_{2}\mathrm{is}}$ (*p* = 0.039). HFmrEF + BAY41 rats had a significantly increased $P_{O_{2}\mathrm{is}}$ during steady‐state contractions in comparison to HFmrEF rats (Figure 3a, blue area; 140–180 s; *p *< 0.05 for each 2 s increment). The differences between the $P_{O_{2}\mathrm{is}}$ profiles during the rest–contraction transient in HFmrEF groups were such that a higher overall muscle oxygenation (AUC) was observed within the spinotrapezius muscle of HFmrEF + BAY41 versus HFmrEF rats (*p* = 0.047).

**TABLE 2:** Interstitial $P_{O_{2}}$ kinetics during 180 s of contractions.

Parameter	Healthy	Healthy + BAY41	*p*‐Value	HFmrEF	HFmrEF + BAY41	*p*‐Value
	(*n *= 8)	(*n *= 8)		(*n *= 7)	(*n *= 10)	
$P_{O_{2}\left(\mathrm{BL}\right)}$, mmHg	26.4 ± 4.2	31.6 ± 3.0*	0.020	22.5 ± 4.6	26.2 ± 2.9*	0.039
$\Delta_{1}P_{O_{2}}$, mmHg	14.4 ± 2.8	15.1 ± 1.6	0.630	12.8 ± 4.3	12.7 ± 4.7	0.999
TD, s	10 ± 4	8 ± 6	0.542	5 ± 3	8 ± 4	0.094
T_63_, s	25 ± 10	25 ± 5	0.974	25 ± 6	26 ± 10	0.967
$P_{O_{2}\left(\mathrm{Nadir}\right)}$, mmHg	12.0 ± 3.6	16.5 ± 3.7*	0.022	9.7 ± 3.1	13.4 ± 4.9	0.096
MRT, s	34 ± 12	35 ± 7	0.751	31 ± 9	34 ± 11	0.529
$\Delta_{2}P_{O_{2}}$, mmHg	1.5 ± 1.0	5.3 ± 3.9*	0.009	3.0 ± 2.2	3.2 ± 2.2	0.919
TD_2_, s	76 ± 43	78 ± 8	0.934	77 ± 29	74 ± 26	0.879
$P_{O_{2}\left(\mathrm{End}-\mathrm{Contract}\right)}$, mmHg	14.2 ± 3.6	20.8 ± 6.2*	0.005	12.4 ± 1.7	16.6 ± 6.3*	0.049
AUC, mmHg·s	1333 ± 362	1826 ± 388*	0.012	1108 ± 239	1461 ± 427*	0.047

*Note*: Data are means ± SD. *n*, number of rats. Abbreviations: AUC, area under the curve during 0–180 s; BAY41, BAY 41‐2272; HFmrEF, heart failure with mildly reduced ejection fraction; $P_{O_{2}\left(\mathrm{BL}\right)}$, $P_{O_{2}}$ at baseline (−30 to 0 s); $P_{O_{2}\left(\mathrm{End}-\mathrm{Contract}\right)}$, $P_{O_{2}}$ at the end contractions (averaged 160–180 s); $\Delta_{1}P_{O_{2}}$, primary amplitude; $P_{O_{2}\left(\mathrm{Nadir}\right)}$, lowest recorded $P_{O_{2}}$ during contractions; MRT, mean response time; $\Delta_{2}P_{O_{2}}$, secondary amplitude; TD, time delay; TD_2_, secondary time delay; *T*_63_, calculated time to reach 63% of the kinetic response. * *p *< 0.05 versus vehicle within group; analysed via two‐way ANOVA with Tukey's *post hoc* analyses.

### 3.5 Sodium nitroprusside interstitial $P_{O_{2}}$ kinetics

**FIGURE 4:**
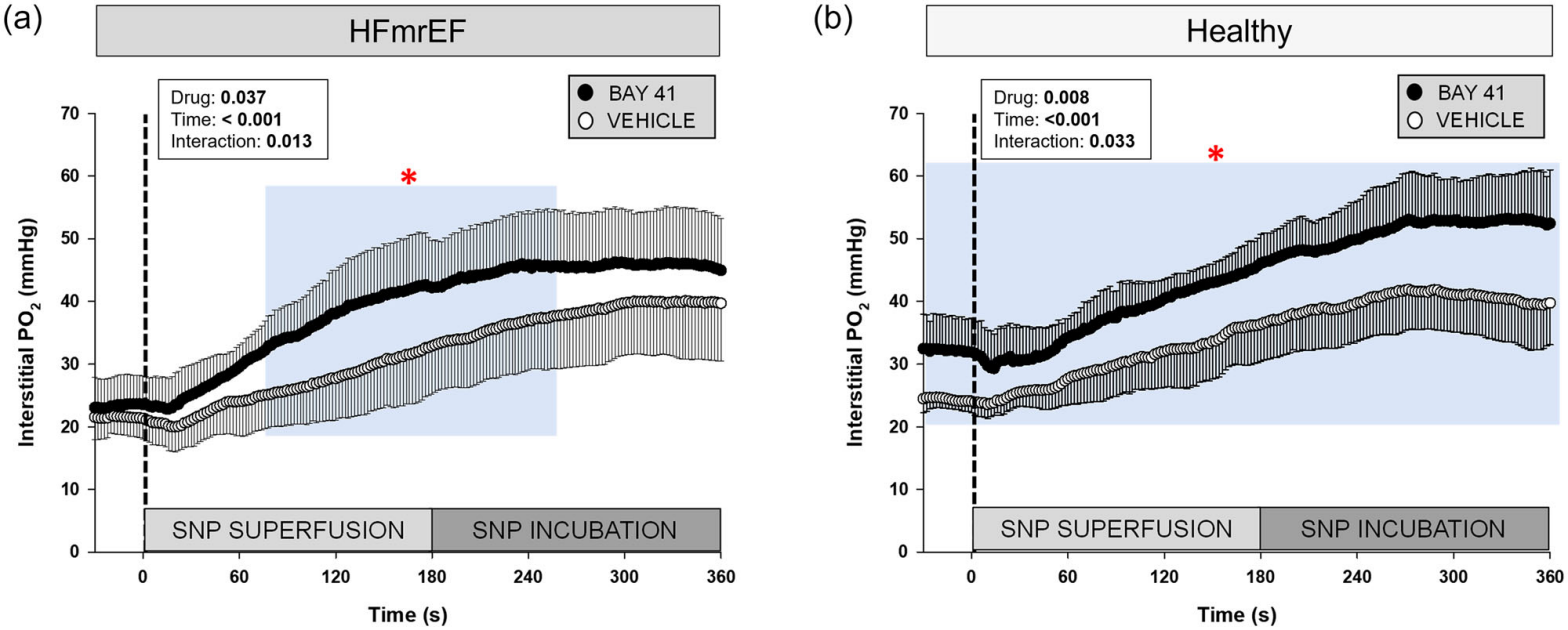
Spinotrapezius muscle interstitial $P_{O_{2}}$ response during SNP superfusion. Data are means ± SD for Healthy rats (*n *= 8), Healthy + BAY41 (*n *= 6), HFmrEF (*n *= 7) and HFmrEF + BAY41 (*n *= 10). At 0 s (dashed line) SNP was continuously superfused onto the muscle. The incubation period reflects a time for the $P_{O_{2}\mathrm{is}}$ response to reach a steady state. (a) HFmrEF + BAY41 rats had a significantly higher $P_{O_{2}\mathrm{is}}$ during 78–262 s (blue area) versus HFmrEF vehicle control rats. There was a significant drug × time interaction for the HFmrEF groups (*p* = 0.013). (b) Healthy + BAY41 rats had a significantly greater $P_{O_{2}\mathrm{is}}$ throughout the entire response. The $P_{O_{2}\mathrm{is}}$ was compared across time via two‐way repeated‐measures ANOVA with Tukey's *post hoc* analyses. *p < 0.05 for each 2 s increment versus Vehicle within group. Abbreviations: BAY41, BAY 41‐2272; HFmrEF, heart failure with mildly reduced ejection fraction; $P_{O_{2}\mathrm{is}}$, interstitial $P_{O_{2}}$ ; SNP, sodium nitroprusside.

**FIGURE 5:**
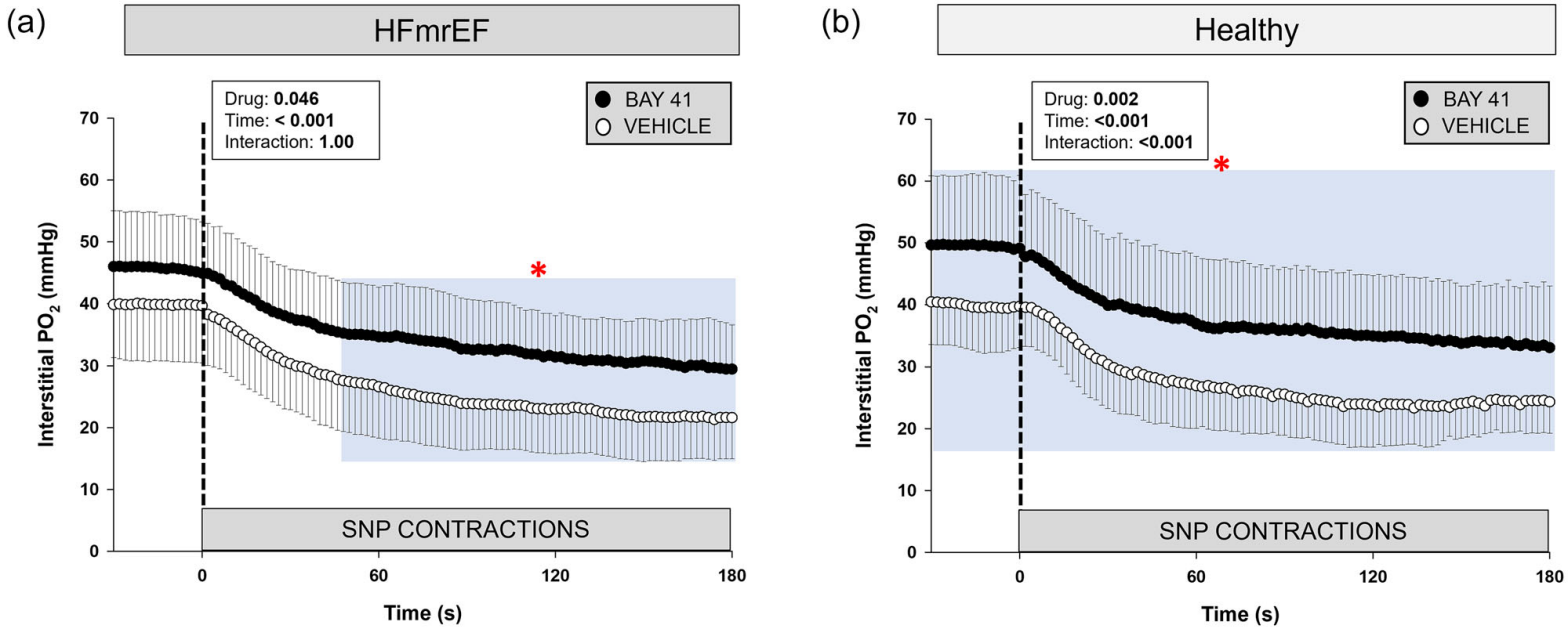
Spinotrapezius muscle interstitial $P_{O_{2}}$ during contractions after SNP superfusion. Data are means ± SD. The dashed line represents the onset of twitch contractions for Healthy rats (*n *= 8), Healthy + BAY41 (*n *= 6), HFmrEF (*n *= 7) and HFmrEF + BAY41 (*n *= 10). (a) HFmrEF + BAY41 rats had a significantly higher $P_{O_{2}\mathrm{is}}$ during 46–180 s (blue area) versus HFmrEF vehicle control rats. (b) Healthy + BAY41 rats had a significantly greater $P_{O_{2}\mathrm{is}}$ throughout the entire response. The $P_{O_{2}\mathrm{is}}$ was compared across time via two‐way repeated‐measures ANOVA with Tukey's *post hoc* analyses. *p < 0.05 for each 2 s increment versus Vehicle within group. Abbreviations: BAY41, BAY 41‐2272; HFmrEF, heart failure with mildly reduced ejection fraction; $P_{O_{2}\mathrm{is}}$, interstitial $P_{O_{2}}$; SNP, sodium nitroprusside.

**FIGURE 6:**
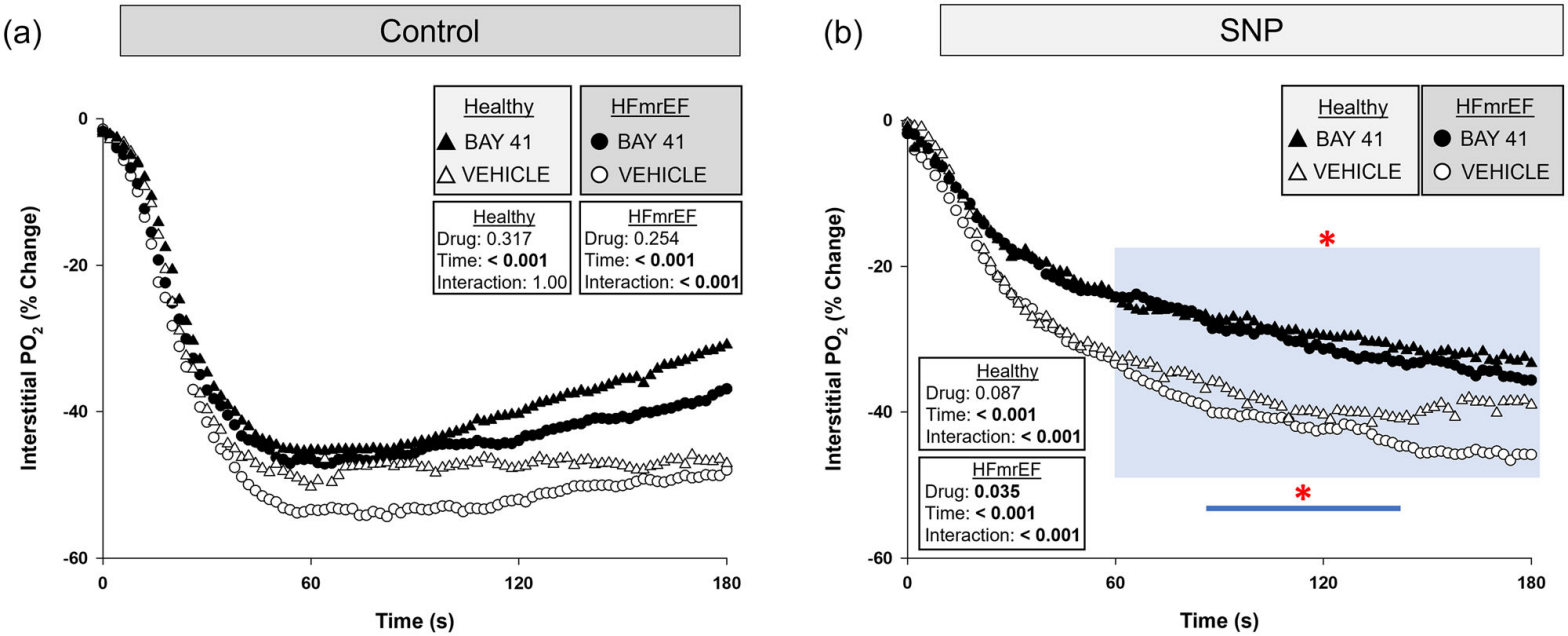
Normalized spinotrapezius muscle interstitial $P_{O_{2}}$ during twitch contractions in both control (CON, no superfusion; a) and SNP superfusion (b) conditions. The onset of contractions is at 0 s. Data are averaged across animals. Control: Healthy (*n *= 8), Healthy + BAY41 (*n *= 8), HFmrEF (*n *= 7) and HFmrEF + BAY41 (*n *= 10); and SNP: Healthy (*n *= 8); Healthy + BAY41 (*n *= 6); HFmrEF (*n *= 7) and HFmrEF + BAY41 (*n *= 10). Error bars are omitted for clarity. (a) There was no significant difference in percentage change within groups during control contraction. (b) SNP slows the kinetic response to contractions in all groups versus control conditions, but to the greatest extent in the BAY41‐treated rats. There was a statistically lower $P_{O_{2}\mathrm{is}}$ percentage change in HFmrEF + BAY41 rats during 60–180 s (blue area) and in Healthy + BAY41 rats from 86 to 142 s (blue line). The $P_{O_{2}\mathrm{is}}$ was compared across time via two‐way repeated‐measures ANOVA with Tukey's *post hoc* analyses. *p < 0.05 for each 2 s increment versus Vehicle within group. Abbreviations: BAY41, BAY 41‐2272; HFmrEF, heart failure with mildly reduced ejection fraction; $P_{O_{2}\mathrm{is}}$, interstitial $P_{O_{2}}$; SNP, sodium nitroprusside.

Thirty‐one rats (Healthy, *n *= 8; Healthy + BAY41, *n *= 6; HFmrEF, *n *= 7; HFmrEF + BAY41, *n *= 10) were analysed for SNP $P_{O_{2}\mathrm{is}}$ kinetics. Two Healthy + BAY41 rats were excluded from the data set owing to haemodynamic complications during SNP superfusion (mean arterial pressure < 70 mmHg) (Behnke et al., 2006). Mean SNP superfusion $P_{O_{2}\mathrm{is}}$ kinetics parameters are presented in Table 3. HFmrEF + BAY41 rats had a greater sensitivity to SNP compared with HFmrEF rats, as evidenced by the reduced *T*_63_ (*p* = 0.011) and MRT (*p* = 0.022). During SNP superfusion and incubation there was a greater increase in $P_{O_{2}\mathrm{is}}$ in HFmrEF + BAY41 in comparison to HFmrEF rats (Figure 4a; 78–262 s; *p *< 0.05 for each 2 s increment) and, as a result, higher overall muscle interstitial oxygenation (i.e., AUC; *p* = 0.033). Mean spinotrapezius muscle $P_{O_{2}\mathrm{is}}$ responses after SNP superfusion and during contractions are shown in Figure 5, and the kinetics parameters derived from model fits are presented in Table 4. SNP elevated $P_{O_{2}\mathrm{is}}$ in HFmrEF + BAY41 versus HFmrEF rats during the steady‐state contractions (46–180 s; Figure 5a, *p *< 0.05 for each 2 s increment). To visualize the different temporal profiles among groups better, Figure 6 illustrates the relative (percentage change from $P_{O_{2}\mathrm{is}}$ at baseline) kinetics during the CON and SNP protocols. Note the greater time constant (slower rate of decline) in all groups following SNP, which occurred to a greater degree in BAY41‐treated rats (Healthy and HFmrEF).

**TABLE 3:** Interstitial $P_{O_{2}}$ kinetics during sodium nitroprusside superfusion.

Parameter	Healthy	Healthy + BAY41	*p*‐Value	HFmrEF	HFmrEF + BAY41	*p*‐Value
	(*n *= 8)	(*n *= 6)		(*n *= 7)	(*n *= 10)	
$P_{O_{2}\left(\mathrm{BL}\right)}$, mmHg	24.6 ± 1.5	31.1 ± 4.0*	0.001	21.3 ± 3.7	23.8 ± 5.7	0.299
$\Delta_{1}P_{O_{2}}$, mmHg	15.3 ± 6.6	21.8 ± 5.3	0.072	18.6 ± 8.4	22.0 ± 9.1	0.427
TD, s	71 ± 57	61 ± 28	0.693	57 ± 39	45 ± 27	0.424
*T*_63_, s	138 ± 74	184 ± 69	0.265	209 ± 38	136 ± 58*	0.011
MRT, s	226 ± 102	245 ± 96	0.749	220 ± 50	150 ± 58*	0.022
$P_{O_{2}\left(\mathrm{End}-\mathrm{SF}\right)}$, mmHg	39.8 ± 6.9	52.8 ± 7.6*	0.006	39.9 ± 9.7	45.8 ± 9.2	0.205
AUC, mmHg·s	6332 ± 981	8025 ± 977*	0.002	5789 ± 1283	7151 ± 1306*	0.033

*Note*: Data are means ± SD. *n*, number of rats. Abbreviations: AUC, area under the curve during 0–180 s; BAY41, BAY 41‐2272; HFmrEF, heart failure with mildly reduced ejection fraction; $P_{O_{2}\left(\mathrm{BL}\right)}$, $P_{O_{2}}$ at baseline (−30 to 0 s); $P_{O_{2}\left(\mathrm{End}-\mathrm{SF}\right)}$, $P_{O_{2}}$ at the end superfusion (averaged 160–180 s); $\Delta_{1}P_{O_{2}}$, primary amplitude; MRT, mean response time; TD, time delay; *T*_63_, calculated time to reach 63% of the kinetic response. *p < 0.05 versus vehicle within group; analysed via two‐way ANOVA with Tukey's *post hoc* analyses.

**TABLE 4:** Interstitial $P_{O_{2}}$ kinetics after sodium nitroprusside superfusion and during 180 s of contractions.

Parameter	Healthy	Healthy + BAY41	*p*‐Value	HFmrEF	HFmrEF + BAY41	*p*‐Value
	(*n *= 8)	(*n *= 6)		(*n *= 7)	(*n *= 10)	
$P_{O_{2}\left(\mathrm{BL}\right)}$, mmHg	39.8 ± 6.8	52.5 ± 7.6*	0.007	39.6 ± 9.5	45.6 ± 9.1	0.189
$\Delta_{1}P_{O_{2}}$, mmHg	15.5 ± 3.5	16.7 ± 6.2	0.653	17.9 ± 6.1	16.3 ± 5.5	0.570
TD, s	4 ± 4	5 ± 5	0.813	4 ± 4	3 ± 4	0.681
*T*_63_, s	30 ± 6	42 ± 8*	0.005	51 ± 25	66 ± 36	0.330
$P_{O_{2}\left(\mathrm{Nadir}\right)}$, mmHg	21.6 ± 6.1	35.3 ± 8.9*	0.005	20.8 ± 8.0	27.1 ± 9.8	0.160
MRT, s	42 ± 16	59 ± 23	0.116	54 ± 25	68 ± 38	0.379
$P_{O_{2}\left(\mathrm{End}-\mathrm{Contract}\right)}$, mmHg	24.3 ± 5.1	35.8 ± 8.5*	0.008	21.6 ± 7.3	29.3 ± 7.2*	0.042
AUC, mmHg·s	2470 ± 580	3630 ± 731*	0.002	2365 ± 682	3104 ± 703*	0.027

*Note*: Data are means ± SD. *n*, number of rats. Abbreviations: AUC, area under the curve during 0–180 s; BAY41, BAY 41‐2272; HFmrEF, heart failure with mildly reduced ejection fraction; MRT, mean response time; $P_{O_{2}\left(\mathrm{BL}\right)}$, $P_{O_{2}}$ at baseline (−30 to 0 s); $P_{O_{2}\left(\mathrm{End}-\mathrm{Contract}\right)}$, $P_{O_{2}}$ at the end contractions (averaged 160–180 s); $P_{O_{2}\left(\mathrm{Nadir}\right)}$, lowest recorded $P_{O_{2}}$ during contractions; $\Delta_{1}P_{O_{2}}$, primary amplitude; TD, time delay; *T*_63_, calculated time to reach 63% of the kinetic response. * *p *< 0.05 versus vehicle within group; analysed via two‐way ANOVA with Tukey's *post hoc* analyses.

### 3.6 Expression of sGCβ1 and SOD1

**FIGURE 7:**
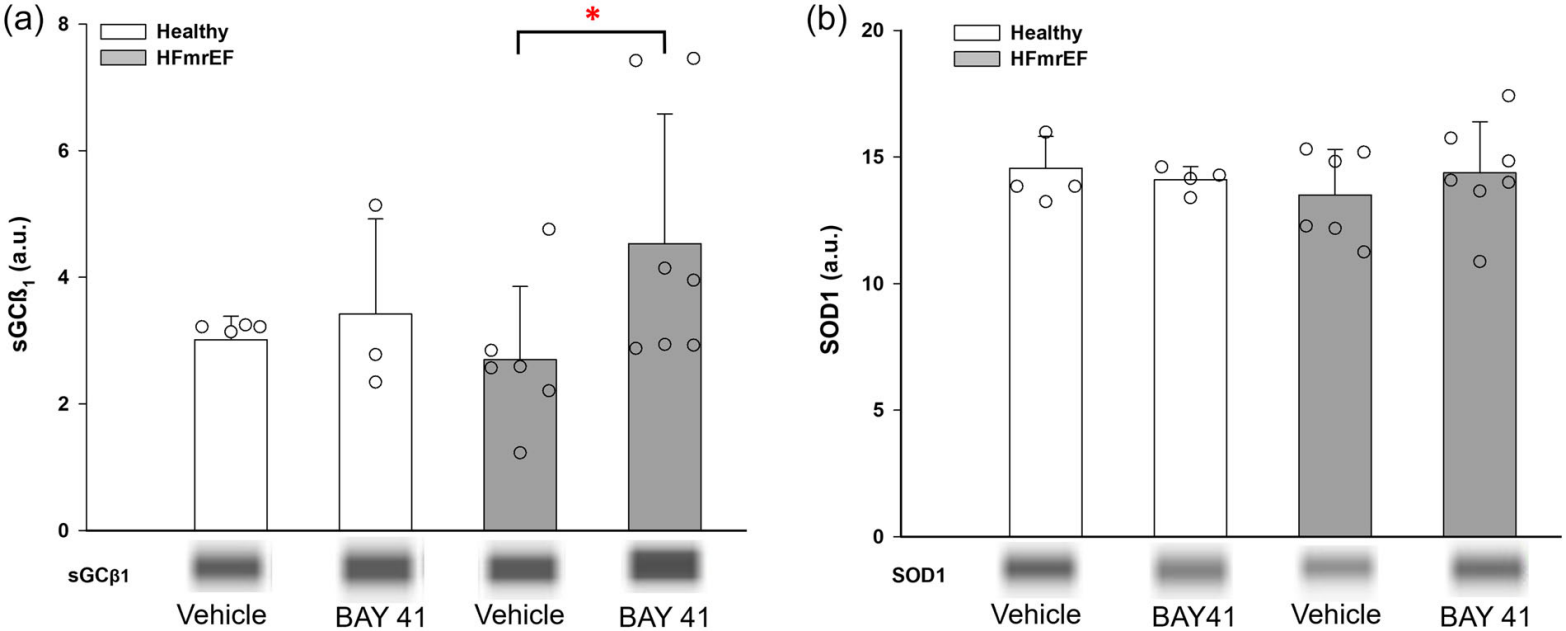
Spinotrapezius muscle sGCβ1 (a) and SOD1 (b) protein expression. Data are means ± SD. Twenty‐one samples of spinotrapezius muscle (Healthy, *n *= 4; Healthy + BAY41, *n *= 4; HFmrEF, *n *= 6; HFmrEF + BAY41, *n *= 7) were used to measure sGCβ1 and SOD1 expression via western immunoblotting. Representative western blot images are presented below each graph. (a) There was a significantly greater expression of sGCβ1 in the spinotrapezius muscle of HFmrEF + BAY41 rats (*p = 0.045). (b) No statistical difference was detected in spinotrapezius muscle SOD1 expression (Healthy, *p* = 0.932; HFmrEF, *p* = 0.897). Data were analysed via two‐way mixed‐effects ANOVA and Fisher's least significant difference test without correction for multiple comparisons. Abbreviations: a.u., arbitrary units; BAY41, BAY 41‐2272; HFmrEF, heart failure with mildly reduced ejection fraction; sGCβ1, soluble guanylyl cyclase beta 1 subunit; SOD1, superoxide dismutase 1.

Twenty‐one samples of spinotrapezius muscle (Healthy, *n *= 4; Healthy + BAY41, *n *= 4; HFmrEF, *n *= 6; HFmrEF + BAY41, *n *= 7) produced clear and quantifiable bands for analysis of sGCβ1 and SOD1 expression via western immunoblotting (Figure 7). There was a significant increase in spinotrapezius muscle sGCβ1 expression in HFmrEF + BAY41 versus HFmrEF vehicle controls (*p* = 0.045), whereas no such differences were detected between Healthy groups (*p* = 0.733). SOD1 protein expression was not different between groups or within conditions (*p *> 0.05).

## 4 DISCUSSION

This is the first investigation, to our knowledge, to demonstrate that a sGC stimulator (BAY 41‐2272) can increase exercise tolerance, potentially by altering the dynamic matching between skeletal muscle ${\dot{Q}}_{O_{2}}$ and ${\dot{V}}_{O_{2}}$ in HFmrEF rats. Specifically, compared with HFmrEF vehicle control rats, BAY41‐treated HFmrEF rats had an elevated $P_{O_{2}\mathrm{is}}$ during steady‐state contractions coupled with an increased responsiveness to SNP superfusion (reduced time constant and increased overall muscle oxygenation). Furthermore, the increased $P_{O_{2}\mathrm{is}}$ and NO sensitivity in HFmrEF + BAY41 rats was associated with an improved exercise tolerance not seen in Healthy rats.

This investigation used the MI model of HF (Pfeffer et al., 1979) to demonstrate characteristics consistent with human pathology. Notably, the HFmrEF condition established herein evidenced a dilated cardiomyopathy (Table 1) with no explicit hypertrophy (unchanged LVPWd, LVPWs or LV mass vs. Healthy rats) or pulmonary congestion (unchanged lung/body weight ratio), which aligns closely with clinical features of intermediate cardiovascular disease in the HFmrEF patient population (Chioncel et al., 2017; Hsu et al., 2017).

### 4.1 Exercise capacity in HFmrEF

Individuals with HFmrEF display an exercise intolerance that is believed to be attributable primarily to impaired O_2_ extraction, similar to HFpEF (Pugliese et al., 2019). However, HFmrEF patients share a phenotype akin to HFrEF (male sex, increased prevalence of ischaemic heart disease and greater benefit from β‐blockers) (Savarese et al., 2022). Despite a lack of effect on peak cardiac output (Pugliese et al., 2019), an MI‐induced redistribution of ${\dot{Q}}_{O_{2}}$ in HFmrEF (Musch & Terrell, 1992) would significantly affect spatial and temporal O_2_ matching (Kindig et al., 1999) within and among skeletal muscles and reduce exercise tolerance preceding cardiac manifestations. We investigated MI rats with an EF of 40%–49% to provide insight into the mechanisms underpinning exercise intolerance in HFmrEF and observed that treatment with an sGC stimulator in HFmrEF rats resulted in a greater (∼40% longer) time to exhaustion in comparison to HFmrEF vehicle‐treated controls (Figure 2). One explanation for this finding is that the sGC stimulator could increase the sensitivity of sGC to the NO–cGMP cascade within fast‐twitch fibres, which are recruited more extensively during exercise in HF than in health (Poole et al., 2012). Altogether, more sensitive NO–sGC signalling would be expected to play a superior role in speeding ${\dot{V}}_{O_{2}}$ kinetics during exercise when mechanically induced endothelial NO synthase activation greatly increases endogenous NO production (Jones et al., 2021).

sGC signalling plays a vital role in skeletal muscle fatigability (Moon et al., 2017) and mitochondrial O_2_ consumption (Hoffmann et al., 2015). Hu et al. (2023) demonstrated, in doxorubicin‐treated mice, that 2 weeks of sGC stimulator administration increased skeletal muscle cGMP content to control levels, which was associated with an increased running time and distance (as seen herein). We found greater sGCβ1 expression in the skeletal muscle of HFmrEF + BAY41 rats (Figure 7). This, coupled with a lack of change in SOD expression, the main antioxidant responsible for superoxide (O_2_·^−^) scavenging (Guzik et al., 2002), suggests reduced O_2_·^−^ production in HFmrEF + BAY41 rats. In rats with arterial hypertension, Ruetten et al. (1999) suggested that early hypertension‐induced changes in sGC signalling can be compensated functionally by sGC agonists, whereas greater disease severity and increased O_2_·^−^ formation render the NO–sGC pathway inert.

### 4.2 Effect of sGC stimulator on interstitial $P_{O_{2}}$ in HFmrEF

Both small (<30%) and large (>30%) MI results in the attenuation and/or redistribution of cardiac output during exercise, which is consistent with peripheral vascular dysfunction and reduced NO bioavailability (Ferreira et al., 2006). sGC stimulators have a dual mode of action, in that they target sGC directly (downstream of endothelial dysfunction, NO scavenging) and increase the sensitivity of sGC to low circulating NO (Sandner et al., 2021). NO–sGC signalling affects skeletal muscle contractile function through both modulation of ${\dot{Q}}_{O_{2}}$ and partial inhibition of cellular respiration (${\dot{V}}_{O_{2}}$) (Jones et al., 2021), which makes sGC a prime candidate to help coordinate skeletal muscle ${\dot{Q}}_{O_{2}}$‐to‐${\dot{V}}_{O_{2}}$ matching (Poole et al., 2012). Such an effect would promote a faster rate of transcapillary O_2_ flux whilst simultaneously lowering the ${\dot{V}}_{O_{2}}$ requirements.

HFmrEF + BAY41 rats had a higher $P_{O_{2}\mathrm{is}}$ during steady‐state contractions versus the HFmrEF vehicle control rats (Figure 3; 140–180 s). There was also an increased overall index of muscle oxygenation within the spinotrapezius muscle of HFmrEF + BAY41 rats during the transition from rest to contractions (0–60 s) and throughout the entire contractile response. When mitochondrial O_2_ consumption increases during contractions, a higher $P_{O_{2}\mathrm{is}}$ is integral to preserve the transmural pressure head necessary for O_2_ diffusion (Hogan et al., 1992). Furthermore, muscular metabolic recovery and phosphocreatine replenishment are dependent on O_2_ availability; therefore, a greater $P_{O_{2}\mathrm{is}}$ at the end of contractions is expected also to reduce fatigue‐accumulating metabolites and increase exercise tolerance (Kindig et al., 2005; Paganini et al., 1997). Interestingly, the HFmrEF + BAY41, HFmrEF and Healthy rats did not have an increased $\Delta_{2}P_{O_{2}}$ (i.e., $P_{O_{2}\mathrm{is}}$ undershoot) like that of Healthy + BAY rats. Figure 6a highlights a substantial $P_{O_{2}\mathrm{is}}$ undershoot in both BAY41‐treated groups compared with the vehicle counterparts when normalizing $P_{O_{2}\mathrm{is}}$. One potential explanation for this finding is that there was a more heterogeneous distribution of ${\dot{Q}}_{O_{2}}$ to the spinotrapezius muscle of Healthy + BAY41 rats, consequent to elevated NO signalling, which might be pertinent to re‐establish the gradient for O_2_ flux into the intracellular compartment.

Acute exposure of smooth muscle cells to sGC stimulators elicits concentration‐dependent relaxation (via increased cGMP production) (Mülsch et al., 1997), which is increased further by exogenous NO (Sandner et al., 2021; Stasch & Hobbs, 2009). Our results are consistent with these findings, given the robust vasodilatory response in BAY41‐treated rats during SNP superfusion (Figure 4). The reduced MRT in HFmrEF + BAY41 rats with SNP superfusion (Table 3) versus HFmrEF vehicle control animals suggests that BAY41 enhances vascular smooth muscle NO responsiveness at rest, which would be expected to compensate for low circulating NO or desensitized sGC in HFmrEF. These findings highlight the mechanistic potential of sGC stimulation to target vascular dysfunction in NO‐deficient states. The SNP contraction response in HFmrEF + BAY41 rats elicited a $P_{O_{2}\mathrm{is}}$ that was ∼2‐fold higher and ∼3‐fold longer (Figure 5a) than that seen during the CON contractions (Figure 3a), which could be attributable, in part, to increased skeletal muscle ${\dot{Q}}_{O_{2}}$ and shear‐stress‐mediated NO production (Katz et al., 1996) that complements the synergistic action of sGC stimulators (Sandner et al., 2021). SNP reduced the incidence and amplitude of the $P_{O_{2}\mathrm{is}}$ undershoot for all groups. The augmented blood flow induced by SNP elevated the nadir $P_{O_{2}\mathrm{is}}$ during contractions to values greater than the steady‐state $P_{O_{2}\mathrm{is}}$ achieved during CON conditions, potentially indicating an excess ${\dot{Q}}_{O_{2}}$ relative to metabolic demand. The blunted $P_{O_{2}\mathrm{is}}$ kinetics response post‐SNP (emphasized in Figure 6) is advantageous to support sustained exercise bouts by preserving the driving pressure that facilitates temporal O_2_ delivery. Earlier reports found that using an sGC activator in moderate HF resulted in a significant increase in the $P_{O_{2}\mathrm{is}}$ during the onset of contractions, without impacting the steady‐state contracting $P_{O_{2}\mathrm{is}}$ (Weber et al., 2022), which contrasts with the sGC stimulator data herein. A higher $P_{O_{2}\mathrm{is}}$ at the onset of contractions is expected to speed ${\dot{V}}_{O_{2}}$ kinetics, reduce the reliance upon phosphagen systems and anaerobic ATP replenishment and decrease intracellular metabolic perturbations that contribute to exhaustion (Poole et al., 2012). Therefore, it is conceivable that the combination of sGC activators and sGC stimulators could effectively target all populations of sGC (haem‐oxidized/haem‐free and wild‐type, native haem) and potentially alter the overall kinetics response.

### 4.3 Clinical implications

Heart failure patients sustain the risk of adverse outcomes even when receiving optimal guideline‐directed medical therapy (Greene et al., 2021), which highlights the importance of investigating the value of novel HF pharmacotherapies across the spectrum of disease severity, especially in the early disease phases (Sato et al., 2017). In HF, the NO–sGC–cGMP pathway has been targeted extensively. However, the efficacy of such an approach might have been hampered by the development of tolerance (e.g., organic nitrate therapy) or dependence on endogenous NO–sGC–cGMP production (e.g., phosphodiesterase inhibitors) (Sandner et al., 2021). Importantly, the mechanism of action of sGC stimulators is preventive in nature (e.g., increased sGC sensitivity to low NO bioavailability). Therefore, sGC stimulators might offer greater benefit to patients with moderate impairments in NO–sGC signalling that occur prior to worsening heart failure. For example, the 2018 Victoria trial evaluated the use of vericiguat (an sGC stimulator) in patients with worsening HFrEF, and *post hoc* analyses revealed that patients with a lower risk of HF progression might have a greater benefit of sGC stimulators (Armstrong et al., 2020; Butler et al., 2022; Lam et al., 2021).

Exercise training is one of the few interventions that improves exercise capacity and ${\dot{V}}_{O_{2}\mathrm{peak}}$ across HF subtypes (O'Connor et al., 2009), reinforcing the importance of skeletal muscle and vascular function in the HF pathophysiology. However, long‐term adherence and accessibility are limiting factors to widespread implementation of structured exercise programmes, particularly in patients experiencing age‐related deficits (frailty, cognitive decline or multiple comorbidities). Small muscle mass exercise training represents a powerful approach to improve convective and diffusive O_2_ transport in HF patients within NYHA class II/III (Esposito et al., 2011) and, in combination with sGC, stimulators might promote enhanced functional outcomes. Patients with chronic thromboembolic pulmonary hypertension who receive treatment with sGC stimulators demonstrate improved exercise capacity and functional outcomes (Ghofrani et al., 2013). Whether this is attributable to improvements in peripheral O_2_ transport remains to be elucidated; however, in preclinical models of pulmonary hypertension, skeletal muscle O_2_ transport is impaired during contractions, owing, in part, to decreased NO signalling (Schulze et al., 2021). Altogether, a medication that directly enhances skeletal muscle O_2_ utilization, vascular function and metabolic efficiency could bridge this gap, offering a much‐needed strategy to improve exercise tolerance and quality of life in HF patients while complementing existing therapies.

### 4.4 Experimental considerations

The MI model of HF in rats permits investigation of physiological mechanisms specific to HFmrEF in the absence of comorbidities and/or multiple pharmaceutical interventions present in human patients. Such factors confound data interpretation and limit physiological insights into experimental treatments. However, it must be emphasized that chronic vascular diseases and risk factors that promote the aetiology of HFmrEF do complicate the translation of these findings to the clinical human population. Moreover, there is a significant difference between the absolute change in EF between healthy and HFmrEF groups herein (∼40%) versus that found in clinical populations (∼25%). This suggests that the HFmrEF rats herein might have greater LV dysfunction than humans with HFmrEF. Notwithstanding this discrepancy, we classified these animals as HFmrEF based on EF and on the lesser degree of myocardial remodelling compared with previous investigations (MI < 40%) and the lack of apparent pulmonary congestion that occurs in congestive HFrEF rats (based on lung‐to‐body weight or LV‐to‐body weight ratio). Collectively, this indicates a more mild–moderate disease state than the severe HFrEF model, where greater impairments in O_2_ transport than those seen in the present investigation have been demonstrated (Ferreira et al., 2006; Musch & Terrell, 1992). Secondly, in consideration of the LV measurements presented, it has been recognized that global longitudinal strain might be a more reliable parameter of LV dysfunction in HF (Kalam et al., 2014) and that EF can change appreciably over time. Herein, we used echocardiographic techniques to assess ventricular function as established previously (Craig et al., 2019) and made repeated measures to best track HFmrEF development. We did not see any improvements in cardiac function with the sGC stimulator in HFmrEF rats. It is important to note that recovery of LV function does not ensure protection from future cardiac events (Merlo et al., 2015). However, measurements such as flow‐mediated dilatation and ${\dot{V}}_{O_{2}\mathrm{peak}}$ represent strong predictors of adverse outcomes independent of EF (Meyer et al., 2005; Pugliese et al., 2019).

## 5 CONCLUSION

The data herein demonstrate that the sGC stimulator BAY 41‐2272 can increase exercise capacity by enhancing skeletal muscle oxygenation in HFmrEF. This effect of pharmacological sGC stimulation on O_2_ transport might be attributable to improvements in vascular function and NO sensitivity in the skeletal muscle vasculature. Investigation of sGC stimulators in combination with exogenous NO‐donors or sGC activators to treat HFmrEF‐related skeletal muscle dysfunction constitutes an exciting avenue for future research.

## AUTHOR CONTRIBUTIONS

Ramona E. Weber, Peter Sandner, David C. Poole and Timothy I. Musch conceived and designed the project; Ramona E. Weber, Kiana M. Schulze, Andrew G. Horn, Tyler E. McCoach, K. Sue Hageman and Zachary J. White performed the experiments; Ramona E. Weber and Zachary J. White completed formal analysis of the data; Ramona E. Weber, Kiana M. Schulze, Andrew G. Horn, Zachary J. White, Stephanie E. Hall, David C. Poole, Timothy I. Musch and Brad J. Behnke interpreted the results of the experiments; Ramona E. Weber prepared the figures and the original draft; all authors edited, revised and approved the final version of this manuscript. All authors agree to be accountable for all aspects of the work in ensuring that questions related to the accuracy or integrity of any part of the work are appropriately investigated and resolved. All persons designated as authors qualify for authorship, and all those who qualify for authorship are listed.

## CONFLICT OF INTEREST

Peter Sandner is an employee of Bayer AG, Pharmaceuticals.

## Data Availability

The data that support the findings of this study are available from the corresponding author upon reasonable request.
